# Identification of clinical and urine biomarkers for uncomplicated urinary tract infection using machine learning algorithms

**DOI:** 10.1038/s41598-019-55523-x

**Published:** 2019-12-23

**Authors:** Amal A. H. Gadalla, Ida M. Friberg, Ann Kift-Morgan, Jingjing Zhang, Matthias Eberl, Nicholas Topley, Ian Weeks, Simone Cuff, Mandy Wootton, Micaela Gal, Gita Parekh, Paul Davis, Clive Gregory, Kerenza Hood, Kathryn Hughes, Christopher Butler, Nick A. Francis

**Affiliations:** 1 Division of Population Medicine, School of Medicine, College of Biomedical and Life Sciences, Cardiff University, Cardiff, United Kingdom; 2 Division of Infection & Immunity, School of Medicine, College of Biomedical and Life Sciences, Cardiff University, Cardiff, United Kingdom; 3 Systems Immunity Research Institute, Cardiff University, Cardiff, United Kingdom; 4 Clinical Innovation Hub, School of Medicine, College of Biomedical and Life Sciences, Cardiff University, Cardiff, United Kingdom; 5 Specialist Antimicrobial Chemotherapy Unit, Public Health Wales Microbiology Cardiff, University Hospital of Wales, Cardiff, United Kingdom; 6 Mologic Ltd., Bedford Technology Park, Thurleigh, Bedford, United Kingdom; 7 Centre for Trials Research, School of Medicine, College of Biomedical and Life Sciences, Cardiff University, Cardiff, United Kingdom; 8 Nuffield Department of Primary Care Health Sciences, University of Oxford, Oxford, United Kingdom; 9 Primary Care, Population Sciences and Medical Education, University of Southampton, Southampton, United Kingdom

**Keywords:** Diagnostic markers, Bacterial infection

## Abstract

Women with uncomplicated urinary tract infection (UTI) symptoms are commonly treated with empirical antibiotics, resulting in overuse of antibiotics, which promotes antimicrobial resistance. Available diagnostic tools are either not cost-effective or diagnostically sub-optimal. Here, we identified clinical and urinary immunological predictors for UTI diagnosis. We explored 17 clinical and 42 immunological potential predictors for bacterial culture among women with uncomplicated UTI symptoms using random forest or support vector machine coupled with recursive feature elimination. Urine cloudiness was the best performing clinical predictor to rule out (negative likelihood ratio [LR−] = 0.4) and rule in (LR+ = 2.6) UTI. Using a more discriminatory scale to assess cloudiness (turbidity) increased the accuracy of UTI prediction further (LR+ = 4.4). Urinary levels of MMP9, NGAL, CXCL8 and IL-1β together had a higher LR+ (6.1) and similar LR− (0.4), compared to cloudiness. Varying the bacterial count thresholds for urine culture positivity did not alter best clinical predictor selection, but did affect the number of immunological predictors required for reaching an optimal prediction. We conclude that urine cloudiness is particularly helpful in ruling out negative UTI cases. The identified urinary biomarkers could be used to develop a point of care test for UTI but require further validation.

## Introduction

Most guidelines for uncomplicated UTI recommend treatment with empirical antibiotics. However, when urine is cultured, approximately only one in three women with UTI symptoms are found to have a UTI as defined by a positive bacterial culture^1^. Therefore, prescribing empirically may result in antibiotic overuse and contribute to development of antimicrobial resistance. Clinicians generally base treatment decisions on symptoms, urine appearance, urine dipstick results, risk factors for development of complications and patient preference^2,3^. Some of these features have been combined into clinical prediction rules, but the predictive values remain suboptimal^4^. Therefore, the development of better diagnostic tools for UTI is essential for improving antimicrobial stewardship.

Exploratory approaches to aid UTI diagnosis have been based on serum and urinary biomarkers. The specificity of blood immune markers is limited by the possibility of cross-reactivity due to other infections or inflammatory responses. Urinary biomarkers that might reflect local immunological responses by the bladder epithelium include nerve growth factor (NGF), chemokines including IL-8/CXCL8^5,6^ and antimicrobial peptides (AMPs), human α-defensin 5 (HD5)^7^ and neutrophil gelatinase-associated lipocalin (NGAL)^8^. However, there is a lack of comprehensive biomarker screening studies for UTI.

With an expansion in the list of potential UTI biomarkers, it is also important to identify the most useful and readily available clinical information that could assist UTI diagnosis and guide prescribing decisions at the point of care. Many studies have implemented multivariate statistical models such as logistic regression to identify UTI clinical predictors^2,4^. These models are bound by relationship assumptions between predictors and outcome variables. In this study we aimed to use a machine learning-based approach, in which random forest (RF) and support vector machines (SVM) were implemented to allow fewer assumptions and more complex relationships between predictors. We combined these algorithms with recursive feature elimination (RFE) to extract the best predictor(s) for uncomplicated UTI using clinical information and potential biomarkers present in urine. These analytical approaches have been widely used in medical applications, such as drug discovery, biomarker selection and early diagnosis^9–16^. SVM, for instance, is a supervised learning model based on statistical learning for classification and regression analysis, which finds the separating hyperplane with the maximal margin between data from different groups. RF is an ensemble learning method that constructs a multitude of decision trees^17^ and is a popular approach for diagnosis^18^ and medical decision support systems^19^. Both SVM and RF outperform other machine learning methods for discriminant problems^20^. In this study, the aim was to find the best biomarker for UTI diagnosis, thus the classification ability was an important factor in differentiating UTI groups. Also, considering the complexity of the raw data required for biomarker discovery, the ability to cope with high-dimensional data was another criterion in choosing machine learning methods. In RF, the trees are decorrelated at each split on a small subset of features rather than all features, thus it is a strong candidate algorithm for high dimensional data. For SVMs, the separate hyperplane relies on the support vectors not all data, thus giving it independent advantages in dealing with high-dimensional data.

## Results

### Clinical information to predict UTI

Our study cohort included 183 women who participated in the POETIC (Point of care testing for urinary tract infection in primary care) trial^21^. They ranged in age from 18 to 85 years, and the key UTI symptoms of urgency, frequency and dysuria were present in 84.2%, 91.8% and 77.0% of patients, respectively. The frequency of other symptoms is presented in Table 1. Following urine culture and according to the POETIC protocol^22^, 79 (43.2%) and 104 (56.8%) patients were classified as UTI positive and negative, respectively. Data from 128 patients (70%) were used for model training while data from 55 patients (30%) were used for testing model performance.

**Table 1:** Frequency of clinical and immunological predictors.

		UTI		
		NO (n = 104)	YES (n = 79)	(n = 183)
**Patient characteristics, symptoms and urine sample appearance**				
		n (%^a^)	n (%^a^)	Total (%^b^)
Age category in years	18–49	69 (64.5)	38 (35.5)	107 (58.5)
	50–64	26 (60.5)	17 (39.5)	43 (23.5)
	>65	9 (27.3)	24 (72.7)	33 (18)
Urgency	Absent	18 (62.1)	11 (37.9)	29 (15.8)
	Present	86 (55.8)	68 (44.2)	154 (84.2)
Frequency	Absent	7 (46.7)	8 (53.3)	15 (8.2)
	Present	97 (57.7)	71 (42.3)	168 (91.8)
Dysuria	Absent	29 (69)	13 (31)	42 (23)
	Present	75 (53.2)	66 (46.8)	141 (77)
Cloudiness	Absent	65 (75.6)	21 (24.4)	86 (47)
	Present	39 (40.2)	58 (59.8)	97 (53)
Turbidity^c^	1	85 (77.3)	25 (22.7)	110 (60.1)
	2	10 (20.4)	39 (79.6)	49 (26.8)
	3	9 (37.5)	15 (62.5)	24 (13.1)
Severity of fever^d^	0	63 (59.4)	43 (40.6)	106 (57.9)
	1	5 (35.7)	9 (64.3)	14 (7.7)
	2	15 (62.5)	9 (37.5)	24 (13.1)
	3	12 (54.5)	10 (45.5)	22 (12)
	4	5 (38.5)	8 (61.5)	13 (7.1)
	5	4 (100)	0 (0)	4 (2.2)
Severity of pain on the sides	0	48 (57.8)	35 (42.2)	83 (45.4)
	1	7 (70)	3 (30)	10 (5.5)
	2	15 (60)	10 (40)	25 (13.7)
	3	9 (45)	11 (55)	20 (10.9)
	4	8 (47.1)	9 (52.9)	17 (9.3)
	5	15 (68.2)	7 (31.8)	22 (12)
	6	2 (33.3)	4 (66.7)	6 (3.3)
Severity of blood in urine	0	78 (55.7)	62 (44.3)	140 (76.5)
	1	4 (66.7)	2 (33.3)	6 (3.3)
	2	10 (66.7)	5 (33.3)	15 (8.2)
	3	8 (66.7)	4 (33.3)	12 (6.6)
	4	1 (25)	3 (75)	4 (2.2)
	5	3 (50)	3 (50)	6 (3.3)
Severity of urine foul smell	0	44 (62.9)	26 (37.1)	70 (38.3)
	1	6 (60)	4 (40)	10 (5.5)
	2	18 (60)	12 (40)	30 (16.4)
	3	20 (66.7)	10 (33.3)	30 (16.4)
	4	8 (42.1)	11 (57.9)	19 (10.4)
	5	5 (55.6)	4 (44.4)	9 (4.9)
	6	3 (20)	12 (80)	15 (8.2)
Severity of dysuria	0	34 (69.4)	15 (30.6)	49 (26.8)
	1	4 (66.7)	2 (33.3)	6 (3.3)
	2	8 (40)	12 (60)	20 (10.9)
	3	18 (64.3)	10 (35.7)	28 (15.3)
	4	11 (44)	14 (56)	25 (13.7)
	5	19 (57.6)	14 (42.4)	33 (18)
	6	10 (45.5)	12 (54.5)	22 (12)
Severity of urgency	0	13 (68.4)	6 (31.6)	19 (10.4)
	1	5 (71.4)	2 (28.6)	7 (3.8)
	2	9 (45)	11 (55)	20 (10.9)
	3	22 (61.1)	14 (38.9)	36 (19.7)
	4	21 (60)	14 (40)	35 (19.1)
	5	20 (55.6)	16 (44.4)	36 (19.7)
	6	14 (46.7)	16 (53.3)	30 (16.4)
Severity of day time frequency	0	9 (60)	6 (40)	15 (8.2)
	1	1 (20)	4 (80)	5 (2.7)
	2	10 (62.5)	6 (37.5)	16 (8.7)
	3	21 (58.3)	15 (41.7)	36 (19.7)
	4	25 (58.1)	18 (41.9)	43 (23.5)
	5	23 (60.5)	15 (39.5)	38 (20.8)
	6	15 (50)	15 (50)	30 (16.4)
Severity of night time frequency	0	20 (57.1)	15 (42.9)	35 (19.1)
	1	11 (91.7)	1 (8.3)	12 (6.6)
	2	12 (70.6)	5 (29.4)	17 (9.3)
	3	20 (52.6)	18 (47.4)	38 (20.8)
	4	17 (50)	17 (50)	34 (18.6)
	5	16 (69.6)	7 (30.4)	23 (12.6)
	6	8 (33.3)	16 (66.7)	24 (13.1)
Severity of abdominal pain	0	33 (55.9)	26 (44.1)	59 (32.2)
	1	7 (41.2)	10 (58.8)	17 (9.3)
	2	15 (75)	5 (25)	20 (10.9)
	3	25 (56.8)	19 (43.2)	44 (24)
	4	14 (58.3)	10 (41.7)	24 (13.1)
	5	7 (50)	7 (50)	14 (7.7)
	6	3 (60)	2 (40)	5 (2.7)
Severity of restricted activity	0	40 (58)	29 (42)	69 (37.7)
	1	12 (63.2)	7 (36.8)	19 (10.4)
	2	12 (63.2)	7 (36.8)	19 (10.4)
	3	18 (60)	12 (40)	30 (16.4)
	4	10 (43.5)	13 (56.5)	23 (12.6)
	5	9 (60)	6 (40)	15 (8.2)
	6	3 (37.5)	5 (62.5)	8 (4.4)
Severity of feeling generally unwell	0	29 (65.9)	15 (34.1)	44 (24)
	1	10 (52.6)	9 (47.4)	19 (10.4)
	2	21 (63.6)	12 (36.4)	33 (18)
	3	23 (63.9)	13 (36.1)	36 (19.7)
	4	12 (42.9)	16 (57.1)	28 (15.3)
	5	6 (46.2)	7 (53.8)	13 (7.1)
	6	3 (30)	7 (70)	10 (5.5)
		Median (Min- Max)	Median (Min- Max)	
TEMP		36.7 (35.2–39.1)	36.7 (35.3–38)	
**Immunological markers**^**e**^				
IL-1α		0.9 (0–2.4)	1.4 (0.2–3.4)	
IL-1β		1.1 (0–2.5)	1.9 (0–3.1)	
IL-2		0.2 (0–2)	0.4 (0–1.7)	
IL-4		0.1 (0–0.4)	0.1 (0–0.6)	
IL-5		0 (0–0.4)	0.1 (0–0.4)	
IL-6		0.5 (0–2.7)	1.1 (0–3.1)	
sIL-6R		2.8 (0–3.9)	3.1 (0–3.9)	
IL-7		0.3 (0–1.2)	0.4 (0–1.4)	
IL-10		0.1 (0–0.8)	0.2 (0–1.1)	
IL-12p70		0.2 (0–0.7)	0.3 (0–1)	
IL-12p40		0.1 (0–1.2)	0.5 (0–1.9)	
IL-13		0.7 (0–1.6)	1 (0–2)	
IL-15		0.1 (0–0.8)	0.2 (0–1.6)	
IL-16		0.6 (0–2.7)	1.7 (0–3.8)	
IL-17A		0.2 (0–4.2)	1.4 (0–4.2)	
IFN-γ		0.5 (0–2)	0.7 (0–1.8)	
GM-CSF		0.2 (0–2.5)	0.4 (0–2.5)	
TNF-α		0.1 (0–1.5)	0.4 (0–2.3)	
TNF-β		0 (0–0.3)	0 (0–0.7)	
CCL2		2 (0.6–3.4)	2.4 (0.6–3.6)	
CCL3		0.8 (0–2.3)	1.2 (0–3.2)	
CCL4		1.1 (0–3)	1.5 (0–3.5)	
CCL11		1 (0–2.3)	1.2 (0–2.6)	
CCL13		0.9 (0–2.1)	1.1 (0–2)	
CCL17		0.5 (0–1.8)	1 (0–2.8)	
CCL22		1.2 (0–2.5)	1.8 (0–3.1)	
CCL26		0.8 (0–1.8)	0.9 (0–2.2)	
CXCL8		2 (0–3.9)	3.1 (0.8–4.6)	
CXCL10		1.1 (0–4.3)	2.4 (0–4.7)	
Creatinine		8.6 (0–9.6)	8.8 (4.1–9.5)	
Cystatin C		4.4 (0–5.4)	4.6 (0–5.4)	
Desmosine		4.7 (0–6.1)	4.8 (3.1–6.5)	
Fibrinogen		4.2 (0–5.5)	5.2 (0–5.6)	
FMLP		3.7 (0–4.4)	3.9 (2.7–4.3)	
HNE		5 (0–6.4)	5.8 (0–6.5)	
HAS		6.7 (0–7.4)	7.3 (5.4–7.5)	
MMP8		0 (0–5.6)	4.6 (0–5.6)	
MMP9		0.9 (0–5.1)	2.3 (0–5.3)	
NGAL		4.5 (0–5.9)	5.5 (0–5.9)	
Ac-PGP		5.6 (0–6.5)	5.6 (0–6.3)	
RBP4		4.9 (0–6)	5.1 (0–6.3)	
VEGF		2 (1.1–3.2)	2.4 (1.3–3.4)	

^a^Percentage out of row total.

^b^Percentage out of total patients (183).

^c^1 = clear or slightly turbid, 2 = moderately turbid and 3 = very turbid.

^d^Severity of symptoms measured on a scale from 0 (not affected) to 6 (as bad as possible). Please note that none of the patients reported a score of 6 for severity of fever and severity of blood in urine

^e^Measured in Pg/ml and values was transformed to log2.

IL: interleukin.

CC or CXC: chemokines.

IFN-γ: interferon-γ.

TNF: tumor necrosis factor.

GM-CSF: granulocyte-macrophage colony-stimulating factor.

VEGF: vascular endothelial growth factor.

MMP: matrix metalloproteinase.

HNE: human neutrophil elastase.

RBP4: retinol binding protein 4.

NGAL: neutrophil gelatinase-associated lipocalin.

HSA: Human Serum Albumin.

FMLP: N-Formylmethionine-leucyl-phenylalanine.

Ac-PGP: N-acetyl Proline-Glycine-Proline.

Using only the clinical data recorded during the initial consultation, urine cloudiness was the best clinical predictor for UTI with an area under the ROC curve (AUC) of 0.72 (95% CI 0.60–0.85), positive predictive value (PPV) 0.65, negative predictive value (NPV) 0.79, positive likelihood ratio (LR+) 2.55, negative likelihood ratio (LR−) 0.37 and F1 score of 0.69 on the test data subset (Table 2). We then substituted cloudiness (measured as a binary yes/no) with a more discriminatory assessment of cloudiness (turbidity score with three categories; Table 1). This substitution resulted in a similar AUC of 0.73 (95% CI 0.60–0.85) and improved PPV 0.76 and LR + 4.38 (Table 2). No other clinical features or age added to the predictive value of cloudiness/turbidity. RF and SVM algorithms produced similar results, except that SVM selected age plus turbidity (Table 2).

**Table 2:** Performance of selection and merged models on test data subset.

Data set	Algorithm	AUC	PPV	NPV	LR+	LR-	F1 score^1^	Selected predictors
**POETIC UTI classification, UTI prevalence 42.6%**								
Clinical markers with cloudiness	RF + RFE	0.72 (0.60–0.85)^2^	0.65 (0.44–0.82)	0.79 (0.59–0.91)	2.55 (1.4–4.6)	0.37 (0.18–0.75)	0.69 (0.57–0.81)	Cloudiness
Clinical markers with turbidity	RF + RFE	0.73 (0.60–0.85)	0.76 (0.50–0.92)	0.73 (0.56–0.86)	**4.38** (1.6–11.7)	0.49 (0.31–0.80)	0.65 (0.52–0.78)	Turbidity
Immunological markers	RF + RFE	0.82 (0.69–0.94)	0.68 (0.45–0.85)	0.75 (0.56–0.88)	2.88 (1.41–5.92)	0.45 (0.25–0.80)	0.67 (0.54–0.80)	IL-1β and MMP9
Selected clinical with cloudiness + selected immunological markers	RF	0.82 (0.70–0.95)	0.75 (0.51–0.90)	0.76 (0.58–0.89)	3.82 (1.72–9.52)	0.42 (0.23–0.73)	0.70 (0.58–0.82)	Cloudiness, IL-1β and MMP9
Selected clinical with turbidity + selected immunological markers	RF	0.76 (0.63–0.90)	0.67 (0.43–0.85)	0.73 (0.54–0.86)	2.61 (1.30–5.59)	0.52 (0.30–0.86)	0.64 (0.51–0.77)	Turbidity, IL-1β and MMP9
Clinical markers with cloudiness	SVM + RFE	0.73 (0.61–0.85)	0.65 (0.44–0.82)	0.79 (0.59–0.91)	2.55 (1.4–4.6)	**0.37 (0.18–0.75)**	0.69 (0.57–0.81)	Cloudiness
Clinical markers with turbidity	SVM + RFE	0.86 (0.76–0.96)	0.76 (0.50–0.92)	0.73 (0.56–0.86)	4.38 (1.6–11.7)	0.49 (0.31–0.80)	0.65 (0.52–0.78)	Turbidity and age category
Immunological markers	SVM + RFE	0.81 (0.68–0.94)	0.82 (0.55–0.95)	0.76 (0.58–0.88)	**6.29** (2.04–19.36)	0.43 (0.26–0.73)	0.70 (0.58–0.82)	MMP9, NGAL, IL-8/CXCL8 and IL-1β
Selected clinical with cloudiness + selected immunological markers	SVM	0.82 (0.70–0.94)	0.79 (0.54–0.93)	0.77 (0.59–0.89)	5.00 (1.93–13.23)	0.40 (0.23–0.71)	0.71 (0.58–0.83)	Cloudiness, MMP9, NGAL, IL-8/CXCL8 and IL-1β
Selected clinical with turbidity + selected immunological markers	SVM	0.79 (0.66–0.92)	0.70 (0.47–0.86)	0.77 (0.58–0.90)	3.04 (1.52–6.24)	0.39 (0.31–0.74)	0.70 (0.58–0.82)	Turbidity, age category, MMP9, NGAL, IL-8/CXCL8 and IL-1β

^1^F1 score: harmonic mean of precision and recall.

^2^95% confidence interval of the performance metric.

AUC: Area under the curve.

PPV: Positive predictive value.

NPP: Negative predictive value.

LR+ and LR−: positive and negative likelihood ratio.

SVM: support vector machine.

RF: random forest.

RFE: recursive feature elimination.

IL: interleukin.

MMP: matrix metalloproteinase.

NGAL: neutrophil gelatinase-associated lipocalin.

CXCL: the chemokine (C-X-C motif) ligand.

### Urinary biomarkers to predict UTI

We previously reported correlations between bacterial infection and defined immune signatures (‘immune fingerprints’) in other scenarios^23,24^. To apply this knowledge to the diagnosis of uncomplicated UTI we conducted a comprehensive analysis of 42 inflammatory biomarkers in urine samples. In line with earlier observations, we found positive correlations between many of the immunological biomarkers measured (Supplementary Figure S1). As a consequence, RFE was employed to select the best biomarkers for predicting UTI. Using the RFE coupled with RF algorithm (RF + RFE), IL-1β and MMP9 were selected as the best predictors with AUC of 0.82 (95% CI 0.69–0.94) and F1 score of 0.67 on the test data subset (Table 2). The diagnostic relevance of IL-1β and MMP9 was corroborated in an independent analysis using the SVM + RFE algorithm, which resulted in the selection of the same urinary biomarkers alongside NGAL and IL-8/CXCL8, with a similar AUC and improved LR+ and F1 score, compared to the RF + RFE selection (Table 2). Adding the selected immunological biomarkers to the model with clinical features (including cloudiness or turbidity) did not improve the predictive properties (Table 2). We conclude that while urine cloudiness was the most useful clinical predictor to rule out negative cases, urinary biomarkers were particularly helpful to predict the presence of UTI in symptomatic women.

### Variable UTI classification guidelines

Finally, we explored whether changing the bacterial count threshold (based on different national and European UTI guidelines) would affect the selection of clinical and immunological predictors. Using the Public Health England (PHE) guidelines^25,26^ to interpret urine culture results, 99 (54.1%) and 84 (45.9%) patients were UTI positive and negative, respectively. The European Association of Urology (EAU) guidelines^27^ classed 118 (64.5%) and 65 (35.5%) as positive and negative, respectively.

Cloudiness/turbidity remained the best clinical predictor when using the PHE or EAU definitions of UTI positivity (Supplementary Table S1). However, the selection of immunological markers varied with UTI classification and the type of machine learning algorithm employed. Using PHE classification, the best predicting model included a combination of urine cloudiness and NGAL, which resulted in a LR+ and LR− of 4.94 and 0.25 respectively, and a good F1 score of 0.82 (Table S1). Using the EAU classification, the combination of turbidity, feeling unwell, foul smell in urine, NGAL and MMP9 resulted in a model with the best predictive properties (Table S1).

## Discussion

This is one of the first studies to use machine learning methods to select clinical features and urinary immunological markers to predict culture results for uncomplicated UTI in primary care. We found that cloudiness of urine samples was the best clinical predictor of microbiologically confirmed UTI among symptomatic women, and that assessing cloudiness using a categorical turbidity scale improved the predictive properties further, particularly in identifying positive UTI. We identified a set of four urinary immunological markers (MMP9, NGAL, IL-8/CXCL8 and IL-1β), which performed slightly better than cloudiness/turbidity when used independently. Changing the definition of UTI positivity to that used by PHE and the EAU standards, and using both RF and SVM algorithms, resulted in some changes to predictors, but urine cloudiness/turbidity, and the immunological markers MMP9, IL-1β and NGAL continued to be important predictors, thereby confirming their relevance in UTI diagnosis.

While normal urine samples are usually clear, white blood cells (WBCs), red blood cells, epithelial cells, proteins, crystals, drugs and microorganisms can cause the urine to become cloudy. In uncomplicated UTI, the presence of WBCs and/or bacteria in urine can lead to urine cloudiness. This is consistent with the findings of our study where urine cloudiness/turbidity consistently came out as the best predictor of UTI. This finding is in keeping with previous studies that investigated urine appearance as part of clinical rules to predict UTI in community settings^4^ and catheterized patients^28^.

Visual assessment of urine cloudiness by health care staff is recommended in some guidelines as a step in the process of diagnosing uncomplicated UTI (for example PHE)^29^. Our results highlight the importance of implementing this guideline in ruling out negative UTI cases, which is helpful for antibiotic stewardship activities. Furthermore, the improvement on positive UTI prediction by using a turbidity score, instead of binary cloudiness, indicates that the assessment of the degree of cloudiness could improve the diagnosis of uncomplicated UTI within a consultation. In our study, turbidity scores were assessed by the microbiology laboratory after samples were transported from GP practices by standard post at room temperature. As urine turbidity may decrease or increase with prolonged transportation due to WBC lysis or bacterial growth, respectively, our samples were preserved in boric acid to protect WBCs and prevent bacterial growth during transportation^30,31^. Of note, we found no correlation between transportation time and turbidity score, indicating that boric acid preservation was sufficient to stabilise the samples (data not shown).

Cloudiness has not yet been used in other studies using machine-learning for UTI prediction. Heckerling and colleagues used neural networks with genetic algorithm feature selection to examine 212 women with suspected UTI^32^. While they found that cloudiness was associated with increased LR+, their genetic algorithm did not retain it for the creation of the neural network. It is possible that this reflects differences between neural networks and RF models. Alternatively, it may reflect differences in the cohort, since the ratio of cloudy:clear urines differed significantly between the two cohorts (current study cloudy:clear ratio 1.13:1, Heckerling *et al*. 5.84:1), suggesting an underlying difference in the data informing the model. Taylor *et al*. also recently used machine learning to predict UTI^33^. They employed the XGBoost machine learning approach with 211 clinical variables to develop models predicting UTI in an emergency department setting. These were reduced to 10 variables (including urine analysis WBCs, bacteria, blood and dysuria) based on expert knowledge and literature reviews. While this approach worked well, it is not suitable for use in primary care given the number of recommended predictors. These studies, along with ours, demonstrate the potential of machine learning algorithms to enhance diagnosis. They also show that the context of the model is vitally important for its utility and that models may need to be customised for end users’ settings.

Predictor selection methods provide an advanced statistical tool to identify markers for infectious diseases but have not yet been widely used^24^. Using a RFE method coupled with either RF or SVM enabled us to simultaneously screen 17 clinical and 42 immunological biomarkers to identify predictors of UTI in symptomatic women in primary care. Nevertheless, we acknowledge that the relatively small sample size of our study in relation to the number of screened predictors may result in some instability of estimates and overfitting. While RFE is known to be particularly robust against overfitting^34^, we minimised this risk by using cross-validation in addition to a good hyperparameter search strategy within each model. During cross-validation, the model was trained on the training set and validated on a subset of the training data at each iteration, which ensured the generalization performance of the model for unseen data^35^. Furthermore, the classifier was trained on all possible combinations of features including the full feature set and the best combination of features (depending on the generalization performance of the model through cross validation) was selected as the searching space for the next step. Moreover, models were tested on an unseen test data set, which was randomly split prior to model training, indicating model generalizability to an independent data set.

The most promising immunological biomarkers identified were MMP9, NGAL, IL-8/CXCL8 and IL-1β as selected by SVM + RFE, while RF + RFE selected only IL-1β and MMP9 but with lower LR+ compared to SVM + RFE. In general, RF identifies the strongest predictors while SVM tends to produce stronger models based on a larger number of weaker predictors. The fact that we used two machine learning algorithms for predictors selection increased the confidence in markers that were selected by the two algorithms. There might be a potential for improvement in the future by using ensemble methods other than RF, however given that both RF and SVM found turbidity/cloudiness, MMP9 and IL-1β to be the best predictors of UTI it is likely that these predictors will remain as the most important markers. Ideally, we would be able to verify these as predictors using a large independent cohort, and we encourage further large studies to validate our findings. It is also interesting to note that the identified immunological markers interact with each other during urological infection by restricting bacterial growth and mediating trans-epithelial movement of neutrophils^36^. IL-1β induces renal production of NGAL in mice model experiments^37^, and NGAL modulates MMP9 activity by protecting it from degradation^38^. MMP-9, NGAL and some interleukins, have been previously studied as potential biomarkers for UTI, particularly in infants and children, however, conclusions were contradictory^39–44^.

Urine culture is an imperfect gold standard to identify UTI. Bacterial pathogens may die during transport, may not grow using conventional culture techniques or may be rendered unidentified due to contamination of urine samples during collection. There are also differences in opinion on the threshold used to identify significant growth, reflected in different microbiological guidelines. This has a direct impact on the reported prevalence of the disease and subsequently on the evaluation of new tools for UTI diagnosis. This has been shown in this study, as variable numbers of immunological markers were required to reach the optimum prediction depending on the underlying threshold guidelines applied.

This study involved women who participated in the POETIC trial, and who had excess urine samples available following the microbiological analyses included in the POETIC study protocol. No other selection criteria were applied and therefore this should be a relatively representative sample of women presenting in primary care with UTI. We found a slightly higher prevalence of positive UTI (43%) in our study compared to the full trial population (35%), but this is likely to be a chance finding and is unlikely to affect the generalisability of our results. Unfortunately, we were not able to compare urine cloudiness/turbidity or immunological markers with the point of care urine dipstick most commonly used in primary care, as dipstick results were not recorded in the POETIC trial. However, previous studies with similar uncomplicated UTI inclusion criteria, found that dipsticks predicted UTI culture results with a PPV between 0.63 and 0.94 and NPV between 0.20 and 0.81 depending on the diagnostic rule used (presence of nitrite, leukocytes esterase or both) and urine culture colony count threshold^4,45^. When dipstick results were based on leukocytes esterase results only, the maximum PPV and NPV was 0.86 and 0.72, respectively^45^. In our study, cloudiness achieved a comparable NPV of up to 0.79, while MMP9, NGAL, IL-8/CXCL8 and IL-1β achieved PPV of 0.82.

In conclusion, we found that urine cloudiness was the best clinical predictor of UTI among symptomatic women, and that grading cloudiness using a turbidity score may improve the predictive value further. We also found that MMP9, NGAL, IL-8/CXCL8 and IL-1β in urine may be useful predictors of UTI. These biomarkers could be used to develop a new point of care test for UTI, subject to validation of our findings in a larger population, across different age groups, using freshly collected urine and a stringent determination of cut-off levels for the individual biomarkers.

## Methods

### Patient population and clinical data

Clinical information and urine samples were collected as part of a two-arm randomized controlled trial, POETIC (Trial number: ISRCTN65200697)^21,46^. The current analysis included participants from England and Wales who had excess urine sample following the initial POETIC microbiology experiments. The POETIC study included women who presented in primary care with at least one key UTI symptom (dysuria, urgency and frequency) that had been present for up to 14 days. Exclusion criteria were pregnancy, signs of complicated UTI, current use of antibiotics and functional or anatomical genitourinary tract abnormalities^21,46^. Clinical data were collected by general practitioners (GPs). Main UTI symptoms were recorded as present/absent and on a scale from 0 (not affected) to 6 (as bad as possible) to measure its severity. Severity of other symptoms such as fever, flank or abdominal pain, blood in urine, unpleasant urine smell, restricted activity and feeling unwell were also measured (Table 1). Urine cloudiness (clear/cloudy) was reported by GPs following sample examination.

### Ethics

Informed consent was obtained from each patient involved in the study as part of the POETIC clinical trial (number: ISRCTN65200697). Ethical approval was given by the Research Ethics Committee (REC) For Wales recognised by the United Kingdom Ethics Committee Authority (UKECA), REC reference 12/WA/0394. This study was conducted in accordance with the principles of the Declaration of Helsinki.

### Sample collection, processing and culture

**Figure 1:**
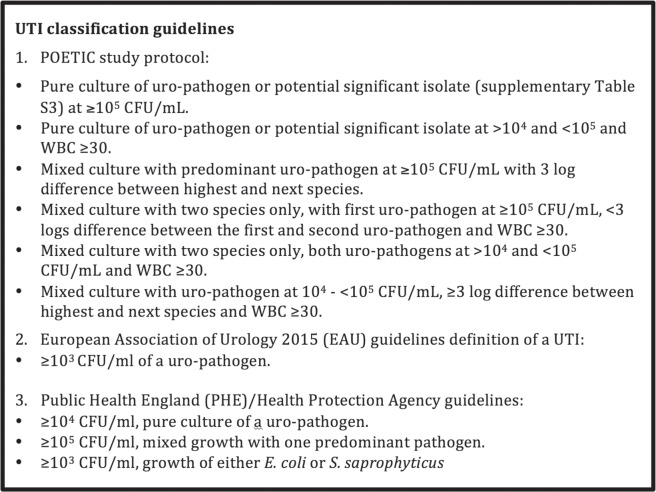
Urinary tract infection definitions according to POETIC study^22^, EAU^27^ and PHE^25,29^.

Mid-stream urine samples were collected at the GP clinic in a universal container containing boric acid and sent to the microbiology laboratory (Specialised Antimicrobial Chemotherapy Unit, University Hospital of Wales, Cardiff) by post. Average time from sample collection to processing in the laboratory was 2.2 [SD = 1.4] days. Urine turbidity was scored by microbiology staff, and for the current analysis, it was categorised as: 1 (clear or slightly turbid), 2 (moderately turbid) and 3 (very turbid). Urine samples were then analysed microscopically and cultured on Columbia Blood Agar (CBA) and CHROMagar UTI Orientation media (E&O) at 34–36 °C for 18–20 hrs^46^. Total and species-specific colony counts were enumerated from CBA and chromogenic agar, respectively. UTI culture positivity was defined as per the POETIC study protocol (Fig. 1).

### Urinary immune biomarker procedure

Cell-free urines were analyzed on a SECTOR Imager 6000 (Meso Scale Discovery) using the V-PLEX Human Cytokine 30-Plex Kit to measure levels of IL-1α, IL-1β, IL-2, IL-4, IL-5, IL-6, IL-7, IL-10, IL-12p40, IL-12p70, IL-13, IL-15, IL-16, IL-17A, IFN-γ, TNF-α, TNF-β, GM-CSF, VEGF, CCL2, CCL3, CCL4, CCL11, CCL13, CCL17, CCL22, CCL26, CXCL8 and CXCL10, and using an ultrasensitive single-plex assays for sIL-6R. Conventional ELISA kits were used to measure creatinine, cystatin C, HSA, MMP8, MMP9 and RBP4 (R&D Systems) as well as fibrinogen (Abcam). HNE was measured using a B.I.T.S. ELISA kit (Mologic); activated PGP, desmosine, FMLP and NGAL were measured using validated in-house developed ELISA kits (Mologic).

### Statistical analysis

#### Data

Our cohort included 183 women with uncomplicated UTI symptoms. For these patients we matched 17 clinical and 70 immunological predictors using patient ID, date of birth and sample ID. There were no missing data on the outcome variable (UTI classes) or the clinical data, however, 28 immunological predictors had missing data of >5% and were therefore removed from the subsequent analysis. Missing data <5% were imputed using Multiple Imputation by Chained Equations in R package “mice” using all variables except the outcome. Imputation methods were predictive mean matching, logistic regression and proportional odds model for numeric variables, binary variables and ordered factor variables, respectively^47^. UTI classes were defined based on the POETIC guidelines^22^ for UTI classification (Fig. 1). Alternative UTI classification guidelines by PHE^25,26^ and the EAU^27^ were used in sensitivity analyses to explore if changing bacterial count threshold for positive UTI would change the marker selection.

#### Analysis approach

**Figure 2:**
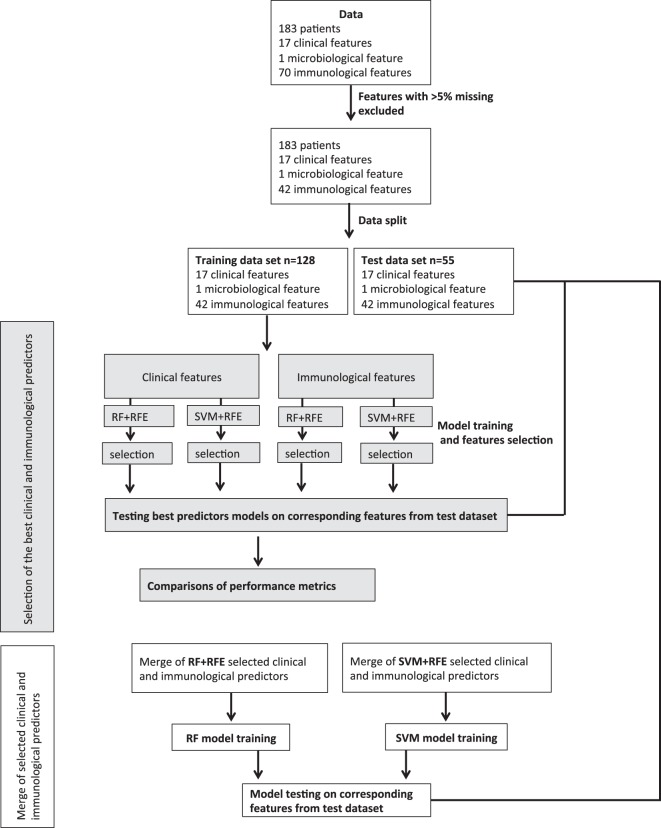
Flowchart of data analysis. RFE: recursive feature elimination. SVM: support vector machine. RF: random forest.

We used the RFE Algorithm 2 on “caret” R package platform^48^, which was coupled with either RF^49^ or SVM (radial basis function kernel in “kernlab” R package)^50^ algorithms to select the best clinical and immunological predictors. RF + RFE and SVM + RFE models were trained on the clinical and immunological predictors separately (Fig. 2). Models were trained on all possible combinations of features including the full feature set and the best combination of features was selected (Supplementary Figure S2). Following the selection of the best clinical and immunological predictors, we aimed to evaluate the additive predictive value of the selected immunological markers on the selected clinical predictors. Thus, we merged the selected clinical and immunological predictors and used them to train RF and SVM models (Fig. 2). Merging the selected clinical and immunological markers was conducted only when a small number of immunological markers were selected.

#### Data pre-processing

For SVM, which does not recognize nominal variables, both binary and ordinal categorical variables were transformed by integer encoding, in which naturally ordered integer numbers were assigned to the levels of the categorical variables to keep the natural order of the clinical data. In addition, continuous data were standardized to a mean of 0 and a variance of 1 for SVM models^51^. For RF models, categorical variables were not transformed because RF can learn directly from categorical data with no data transformation required.

#### Model training and testing

Our data included 183 cases that were randomly split into training (70%) and test (30%) subsets while maintaining the proportion of cases with positive UTI. For all training models, three repeats of 10-fold cross-validation were used to avoid overfitting. During cross-validation, the model was trained on the training set and validated on a subset of the training data at each iteration (cross-validation ROC curves are provided in Supplementary Figure S3). The random search method in the caret package^51^ was implemented to select the optimum hyperparameters (RF: number of features randomly selected for splitting at each tree node [mtry]; SVM: sigma and Cost soft margin [C]; Supplementary Table S2). Model performance was examined on the unseen test data subset. Model performance was compared using the following metrics: AUC, PPV, NPV, LR+, LR−^52^ and F1 Score (harmonic mean of the precision and recall, which range between 0 and 1 where higher value indicates higher performance)^53^. For calculating AUC, the probability threshold for a positive UTI class was set to 0.5. All analyses were performed using R software version 3.4.2^54^.

## Supplementary information

Supplementary Information

## Data Availability

Anonymised clinical and immunological data will be available upon request. The corresponding author or the senior authors (Nick Francis: francisna@cardiff.ac.uk and Chris Butler: christopher.butler@phc.ox.ac.uk) can receive email requests.
