# Supplementary material for: Identification of clinical and urine biomarkers for uncomplicated urinary tract infection using machine learning algorithms
Source: Sci Rep. 2019 Dec 23;9:19694. doi: 10.1038/s41598-019-55523-x (PMC6928162; doi:10.1038/s41598-019-55523-x)
Supplement: Supplementary file 1 — Supplementary Information [file 41598_2019_55523_MOESM1_ESM.pdf]

Supplementary Materials

Identification of clinical and urine biomarkers for uncomplicated urinary tract infection using machine learning algorithms

Amal A. H. Gadalla[1*], Ida M. Friberg[2], Ann Kift-Morgan[2] Jingjing Zhang[2], Matthias Eberl[2,3], Nicholas Topley[2,3], Ian Weeks[3,4], Simone Cuff[2,3,4], Mandy Wootton[5], Micaela Gal[1], Gita Parekh[6], Paul Davis[6], Clive Gregory[1], Kerenza Hood[7], Kathryn Hughes[1], Christopher Butler[1,8†] and Nick A Francis[1,3†]

[1]Division of Population Medicine, School of Medicine, College of Biomedical and Life Sciences, Cardiff University, Cardiff, United Kingdom

[2]Division of Infection & Immunity, School of Medicine, College of Biomedical and Life Sciences, Cardiff University, Cardiff, United Kingdom

[3]Systems Immunity Research Institute, Cardiff University, Cardiff, United Kingdom

[4]Clinical Innovation Hub, School of Medicine, College of Biomedical and Life Sciences, Cardiff University, Cardiff, United Kingdom

[5]Specialist Antimicrobial Chemotherapy Unit, Public Health Wales Microbiology Cardiff, University Hospital of Wales, Cardiff, United Kingdom

[6]Mologic Ltd., Bedford Technology Park, Thurleigh, Bedford, United Kingdom

[7]Centre for Trials Research, School of Medicine, College of Biomedical and Life Sciences, Cardiff University, Cardiff, United Kingdom

[8]Nuffield Department of Primary Care Health Sciences, University of Oxford, Oxford, United Kingdom

[*]**Corresponding author:** GadallaA1@cardiff.ac.uk

[†] joint last authors

Supplementary Materials

Table S1: Performance of selection and merged models on test data subset

Data set	Algorithm	AUC	PPV	NPV	LR+	LR-	F1 score[1]	Selected predictors
**PHE UTI classification, UTI prevalence 53.7%**								
Clinical markers with cloudiness	RF+RFE	0.66 (0.53-0.79)[2]	0.68 (0.49-0.83)	0.65 (0.43-0.83)	1.80 (1.10-3.08)	0.47 (0.24-0.87)	0.70 (0.58-0.82)	Cloudiness
Clinical markers with turbidity	RF+RFE	0.73 (0.61-0.85)	0.77 (0.54-0.91)	0.63 (0.44-0.78)	2.95 (1.26-6.80)	0.51 (0.33-0.81)	0.67 (0.54-0.80)	Turbidity
Immunological markers	RF+RFE	0.83 (0.72-0.94)	0.77 (0.57-0.89)	0.75 (0.53-0.89)	2.82 (1.47-5.46)	0.29 (0.14-0.60)	0.77 (0.66-0.88)	14 biomarkers selected[3]
Selected clinical + selected immunological markers	RF	#						
Clinical markers with cloudiness	SVM+RFE	0.66 (0.53-0.79)	0.68 (0.49-0.83)	0.65 (0.43-0.83)	1.80 (1.10-3.08)	0.47 (0.24-0.87)	0.70 (0.54-0.80)	Cloudiness
Clinical markers with turbidity	SVM+RFE	0.73 (0.61-0.85)	0.77 (0.54-0.91)	0.63 (0.44-0.78)	2.95 (1.26-6.80)	0.51 (0.33-0.81)	0.67 (0.54-0.80)	Turbidity
Immunological markers	SVM+RFE	0.81 (0.69-0.93)	0.79 (0.59-0.91)	0.73 (0.52-0.88)	3.17 (1.53-6.54)	0.32 (0.16-0.62)	0.77 (0.66-0.88)	NGAL
Selected clinical with cloudy+ selected immunological markers	SVM	0.81 (0.68-0.93)	0.85 (0.65-0.95)	0.78 (0.57-0.90)	4.94 (1.98-12.40)	0.25 (0.12-0.51)	0.82 (0.72-0.92)	Cloudiness and NGAL
Selected clinical with turbidity+ selected immunological markers	SVM	0.79 (0.66-0.92)	0.76 (0.56-0.89)	0.72 (0.54-0.87)	2.71 (1.40-5.25)	0.33 (0.17-0.66)	0.76 (0.65-0.87)	Turbidity and NGAL
**EAU UTI classification, UTI prevalence 64.8%**								
Clinical markers with cloudiness	RF+RFE	0.69 (0.55-0.82)	0.79 (0.61-0.91)	0.57 (0.34-0.77)	2.00 (1.08-3.75)	0.41 (0.21-0.76)	0.76 (0.65-0.87)	Cloudiness
Clinical markers with turbidity	RF+RFE	0.68 (0.55-0.81)	0.82 (0.60-0.94)	0.48 (0.31-0.67)	2.57 (1.03-6.49)	0.58 (0.39-0.85)	0.66 (0.53-0.79)	Turbidity

Supplementary Materials

Immunological markers	RF+RFE	0.80 (0.68-0.93)	0.75 (0.58-0.87)	0.64 (0.36-0.86)	1.62 (1.04-2.55)	0.30 (0.12-0.76)	0.80 (0.69-0.91)	NGAL, MMP9, IL-17A, IFN-γ, Fibrinogen, IL-1β, IL-16 and CCL2
Selected clinical + selected immunological markers	RF	#						
Clinical markers with cloudiness	SVM+RFE	0.72 (0.58-0.86)	0.71 (0.55-0.84)	0.58 (0.29-0.83)	1.37 (0.94-1.96)	0.38 (0.14-1.04)	0.78 (0.67-0.89)	Cloudiness and severity of being unwell
Clinical markers with turbidity	SVM+RFE	0.82 (0.70-0.93)	0.77 (0.60-0.88)	0.67 (0.39-0.87)	1.83 (1.11-2.96)	0.26 (0.11-0.67)	0.81 (0.71-0.91)	Turbidity, severity of being unwell and severity of foul smell in urine
Immunological markers	SVM+RFE	0.85 (0.73-0.96)	0.74 (0.59-0.86)	0.73 (0.39-0.93)	1.57 (1.06-2.35)	0.21 (0.06-0.69)	0.82 (0.72-0.92)	NGAL and MMP9
Selected clinical with cloudiness+ selected immunological markers	SVM	0.71 (0.55-0.87)	0.73 (0.57-0.85)	0.70 (0.35-0.92)	1.46 (1.01-2.07)	0.22 (0.07-0.82)	0.81 (0.71-0.91)	Cloudiness, severity of being unwell, NGAL and MMP9
Selected clinical with Turbidity+ selected immunological markers	SVM	0.85 (0.73-0.96)	0.77 (0.61-0.88)	0.82 (0.48-0.97)	1.77 (1.16-2.77)	0.13 (0.03-0.52)	0.85 (0.75-0.95)	Turbidity, severity of being unwell, severity of foul smell in urine, NGAL and MMP9

[1] F1-score: harmonic mean of precision and recall

[2] 95% confidence interval of the performance metric

[3] NGAL, MMP9, IL-1β, HSA, Desmosine, IL-16, CCL3, Fibrinogen, CCL17, IL-17A, MMP8, IL-12p70, IL-8 and CCL13

[#] merging was not considered due to the large number of selected predictors

AUC: Area under the curve

PPV: Positive predictive value

NPP: Negative predictive value

LR+ and LR-: positive and negative likelihood ratio

Supplementary Materials

SVM: Support vector machine

RF: Random Forest

RFE: Recursive Feature Elimination

NGAL: Neutrophil gelatinase-associated lipocalin

MMP: Matrix metalloproteinase

IL: Interleukin

HSA: Human serum albumin

CCL: CC chemokine ligands

IFN-γ: Interferon-γ

Supplementary Materials

Table S2: Hyperparameters for models following predictors' selection. For the selected predictors please refer to Table 2 and Table S1.

Data set	RF: mtry	SVM: sigma	SVM: C	Selected predictors
**POETIC UTI classification, UTI prevalence 42.6%**				
Clinical markers with cloudiness	One predictor			Cloudiness
Clinical markers with turbidity	One predictor			Turbidity
Immunological markers	2			IL-1β and MMP9
Selected clinical with cloudiness + selected immunological markers	2			Cloudiness, IL-1β and MMP9
Selected clinical with turbidity + selected immunological markers	3			Turbidity, IL-1β and MMP9
Clinical markers with cloudiness		0.25	0.25	Cloudiness
Clinical markers with turbidity		0.38	1	Turbidity and age category
Immunological markers		0.66	0.25	MMP9, NGAL, IL-8/CXCL8 and IL-1β
Selected clinical with cloudy+ selected immunological markers		0.51	0.25	Cloudiness, MMP9, NGAL, IL-8/CXCL8 and IL-1β
Selected clinical with turbidity+ selected immunological markers		0.24	0.25	Turbidity, age category, MMP9, NGAL, IL-8/CXCL8 and IL-1β
**PHE UTI classification, UTI prevalence 53.7%**				
Clinical markers with cloudiness	One predictor			Cloudiness

Supplementary Materials

Clinical markers with turbidity	One predictor		Turbidity
Immunological markers	Not taken further		14 biomarkers selected[3]
Selected clinical + selected immunological markers	Not trained		
Clinical markers with cloudiness	0.25	0.25	Cloudiness
Clinical markers with turbidity	0.30	1	Turbidity
Immunological markers	97.6	0.5	NGAL
Selected clinical with cloudiness+ selected immunological markers	21.5	1	Cloudiness and NGAL
Selected clinical with Turbidity+ selected immunological markers	55.4	0.5	Turbidity and NGAL
**EAU UTI classification, UTI prevalence 64.8%**			
Clinical markers with cloudiness	One predictor		Cloudiness
Clinical markers with turbidity	One predictor		Turbidity
Immunological markers	Not taken further		NGAL, MMP9, IL-17A, IFN-γ, Fibrinogen, IL-1β, IL-16 and CCL2
Selected clinical + selected immunological markers	Not trained		
Clinical markers with cloudiness	1.59	1	Cloudiness and severity of being unwell

Supplementary Materials

Clinical markers with turbidity	0.44	1	Turbidity, severity of being unwell and severity of foul smell in urine
Immunological markers	2.27	0.25	NGAL and MMP9
Selected clinical with cloudiness+ selected immunological markers	0.32	0.25	Cloudiness, severity of being unwell, NGAL and MMP9
Selected clinical with Turbidity+ selected immunological markers	0.15	0.25	Turbidity, severity of being unwell, severity of foul smell in urine, NGAL and MMP9

Supplementary Materials

Table S3: Uro-pathogen or potential significant isolate used to define UTI positivity

**Potentially significant isolates**	**Uropathogens**
Enterobacteriaceae (*E. coli, Klebsiella species*)	*E. coli*
Proteus	*Klebsiella* species
Enterococcus species	*Enterobacter* species
Staphylococcus aureus	*Citrobacter* species
Staphylococcus saprophyticus	*Serratia* species
Coagulase negative *Staphylococcus* species	*Morganella* species
Pseudomonas	*Proteus* species
Yeasts	*Pantoea* species
	Group B *Streptococcus* species
	Staphylococcus saprophyticus
	Salmonella species

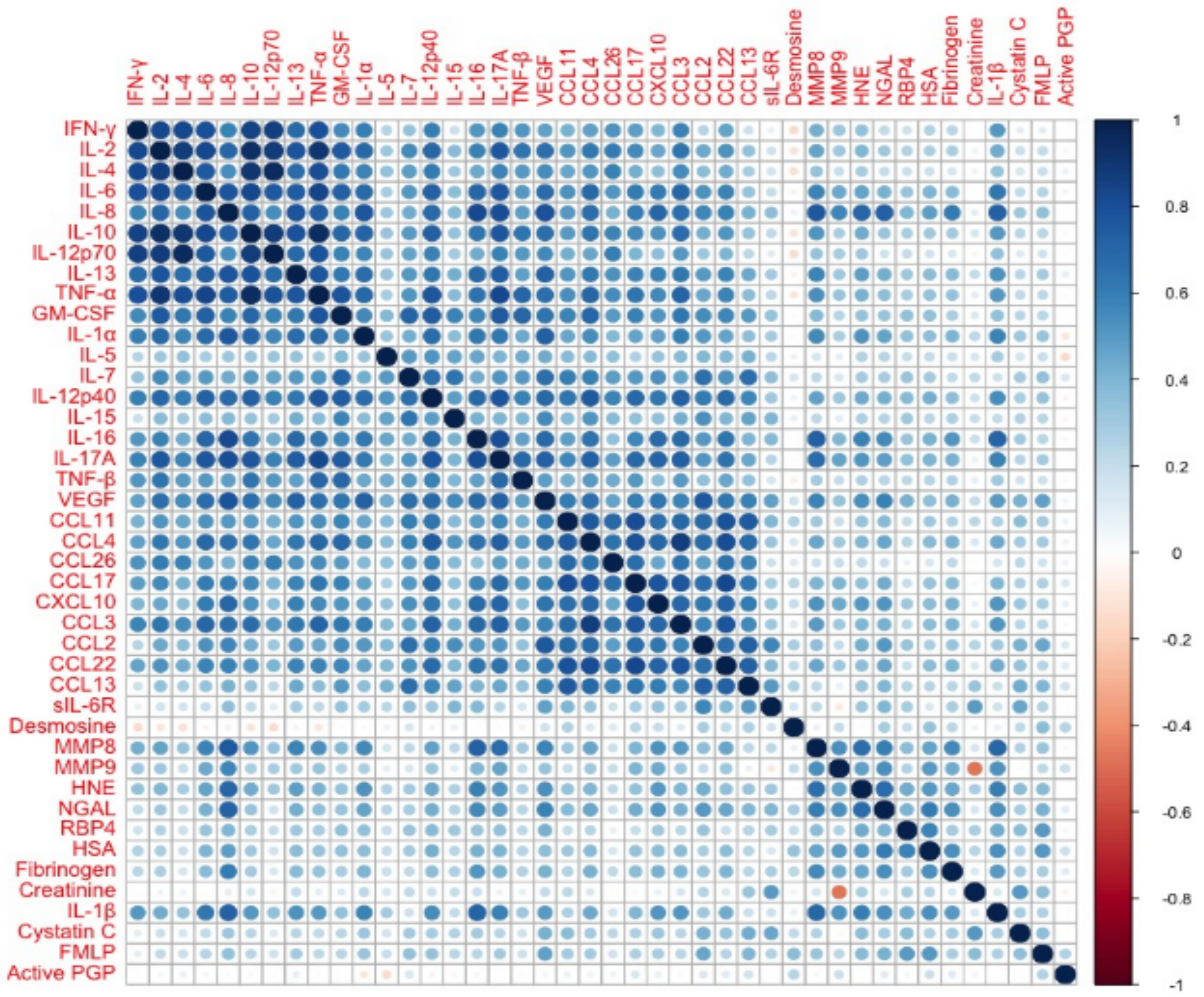

Figure S1: Correlation between immunological markers. Circle size and colour scale represent correlation strength and direction, respectively, between the 42 examined immunological markers. Spearman correlation test was used. R package "corrplot" was used for this visual presentation.

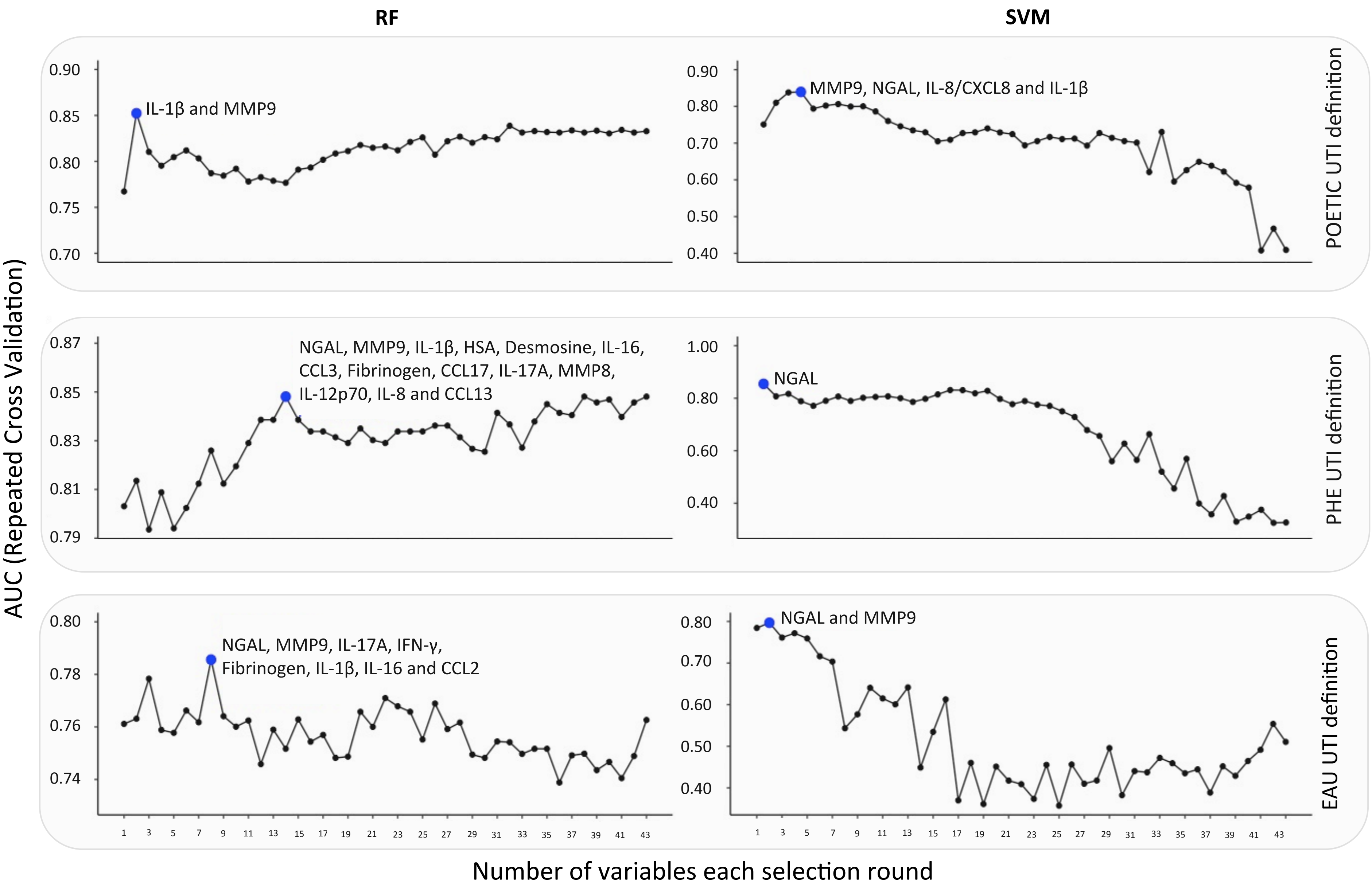

Figure S2: Feature selection among immunological markers using different UTI classification guidelines. POETIC: Point of care testing for urinary tract infection in primary care, PHE: Public Health England, EAU: European Association of Urology, AUC: Area under the ROC curve, RF: Random forest and SVM: Support vector machine

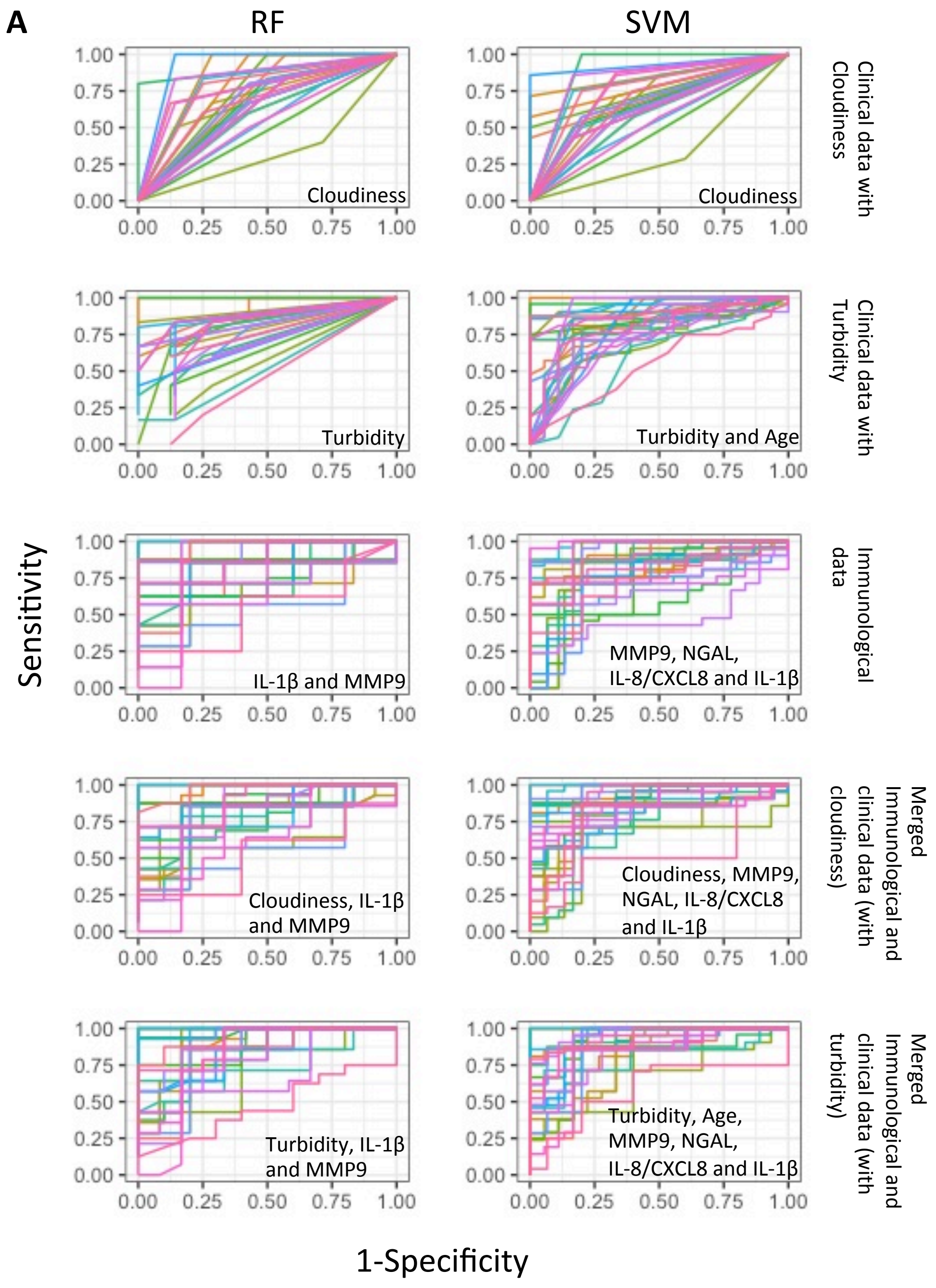

Figure S3: Area under the ROC curve for three repeats of 10-fold cross-validation from models following selection using RF- or SVM- recursive feature elimination. **A.** UTI classified according to the POETIC protocol (Point of care testing for urinary tract infection in primary care trial). **B.** UTI classified according to the Public Health England (PHE) guidelines. **C.** UTI classified according to the European Association of Urology (EAU) guidelines. Predictors included in the model following selection are given in text boxes within each plot. RF: random forest, SVM: support vector machine

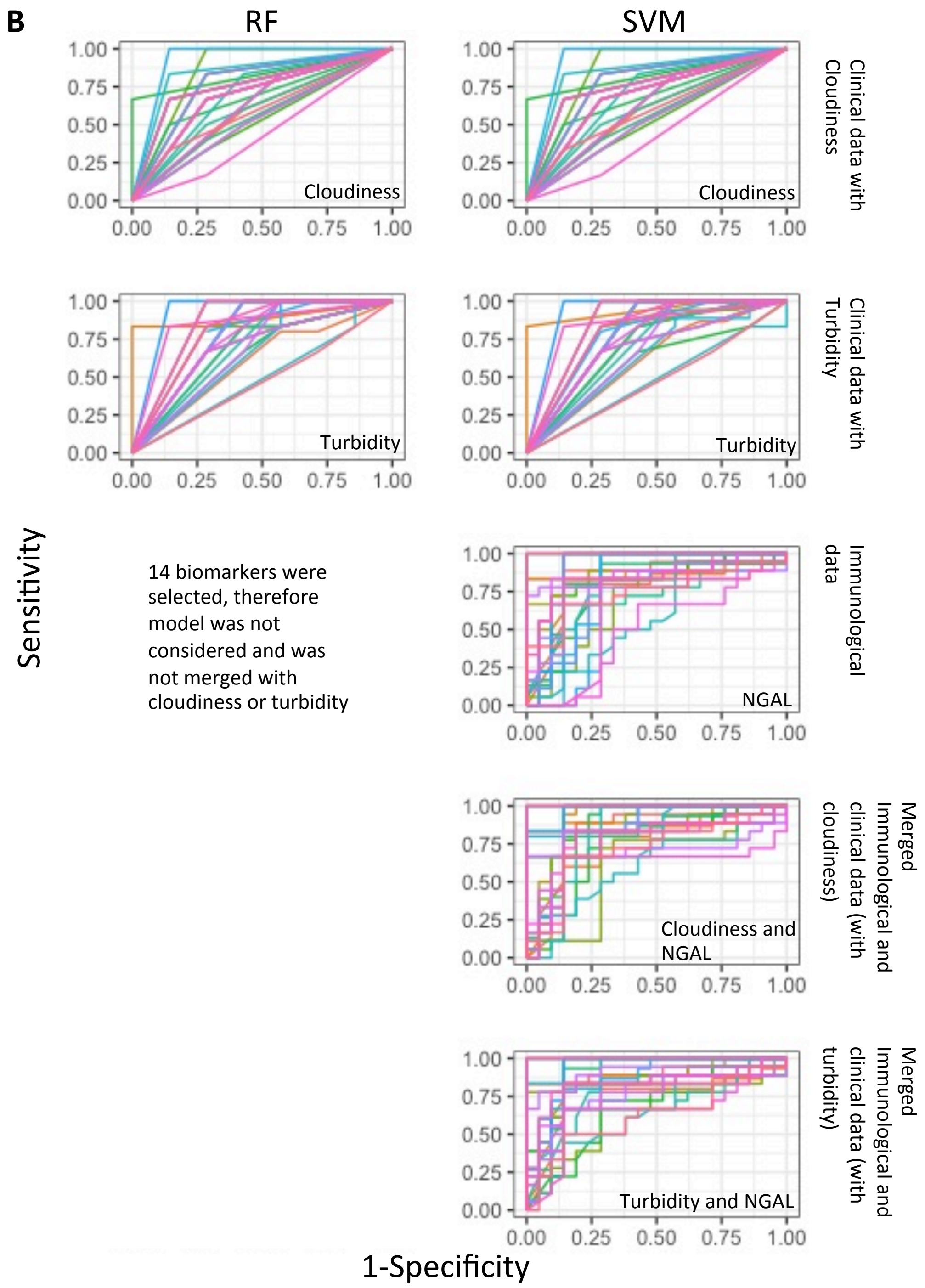
B
RF
SVM
Clinical data with Cloudiness
Clinical data with Turbidity
Immunological data
Merged Immunological and clinical data (with cloudiness)
Merged Immunological and clinical data (with turbidity)
Cloudiness
Turbidity
NGAL
Cloudiness and NGAL
Turbidity and NGAL
14 biomarkers were selected, therefore model was not considered and was not merged with cloudiness or turbidity
Sensitivity
1-Specificity
0.00
0.25
0.50
0.75
1.00

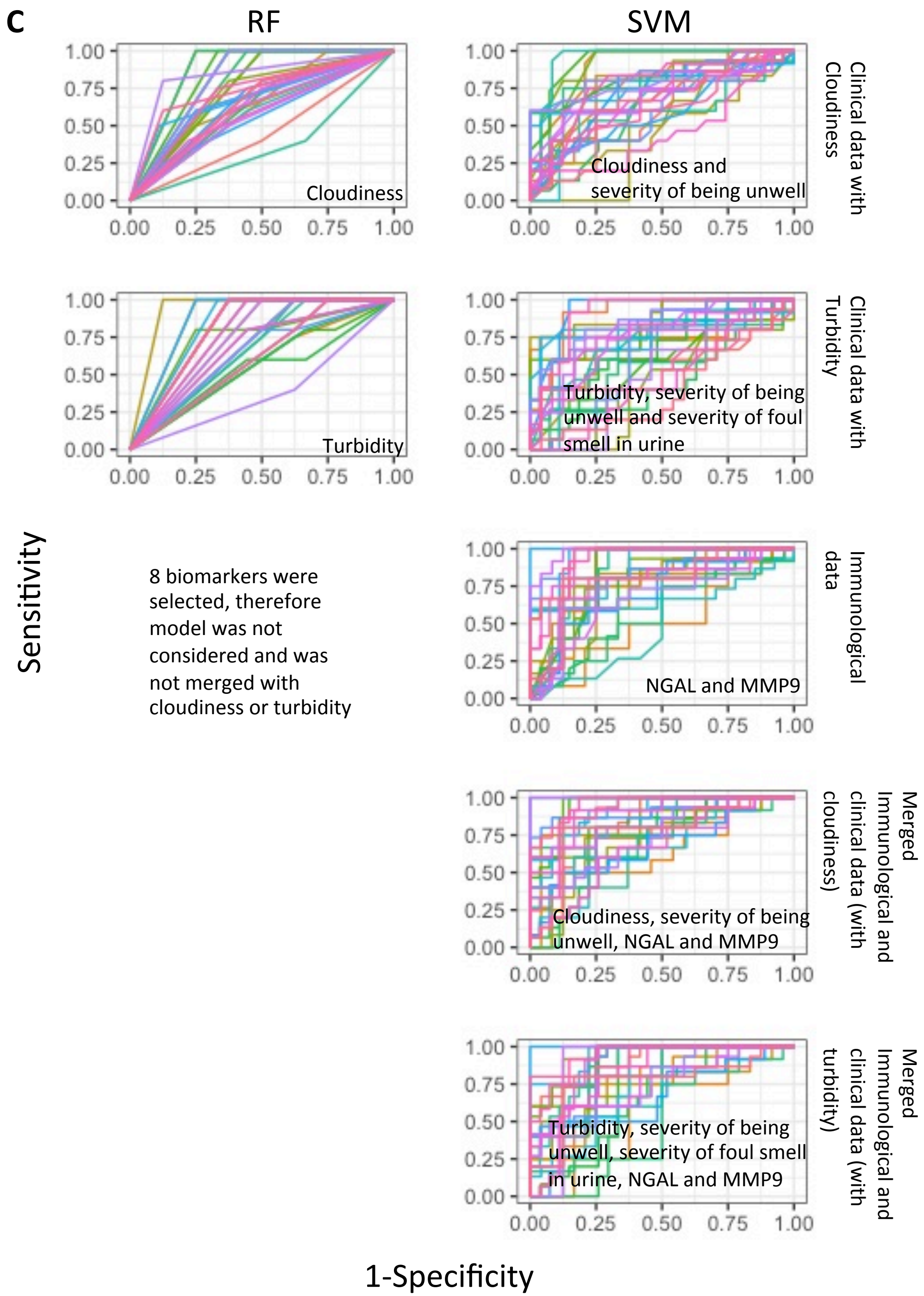
C
RF
SVM
Cloudiness
Cloudiness and severity of being unwell
Clinical data with Cloudiness
Turbidity
Turbidity, severity of being unwell and severity of foul smell in urine
Clinical data with Turbidity
8 biomarkers were selected, therefore model was not considered and was not merged with cloudiness or turbidity
NGAL and MMP9
Immunological data
Cloudiness, severity of being unwell, NGAL and MMP9
Merged Immunological and clinical data (with cloudiness)
Turbidity, severity of being unwell, severity of foul smell in urine, NGAL and MMP9
Merged Immunological and clinical data (with turbidity)
Sensitivity
1-Specificity
0.00
0.25
0.50
0.75
1.00
